# Does the Prostate Health Index Depend on Tumor Volume?—A Study on 196 Patients after Radical Prostatectomy

**DOI:** 10.3390/ijms18030488

**Published:** 2017-02-24

**Authors:** Frank Friedersdorff, Britt Groß, Andreas Maxeiner, Klaus Jung, Kurt Miller, Carsten Stephan, Jonas Busch, Ergin Kilic

**Affiliations:** 1 Department of Urology, Charité University Hospital, 10098 Berlin, Germany; grossbritt@yahoo.de (B.G.); andres.maxeiner@charite.de (A.M.); klaus.jung@charite.de (K.J.); kurt.miller@charite.de (K.M.); carsten.stephan@charite.de (C.S.); Jonas.busch@charite.de (J.B.); 2 Berlin Institute for Urologic Research, 10115 Berlin, Germany; 3 Department of Pathology, Charité University Hospital, 10098 Berlin, Germany; ergin.kilic@charite.de

**Keywords:** prostate cancer, Prostate Health Index, tumor volume

## Abstract

The Prostate Health Index (PHI) has been used increasingly in the context of prostate cancer (PCa) diagnostics since 2010. Previous studies have shown an association between PHI and a tumor volume of >0.5 cm^3^. The aim of this study was to investigate the correlation between PHI and tumor volume as well as the Gleason score. A total of 196 selected patients with prostate cancer treated with radical prostatectomy at our institution were included in our study. The tumor volume was calculated and preoperative serum parameters total prostate-specific antigen (tPSA), free PSA (fPSA), [−2]proPSA, and PHI were evaluated. The association between the pathological findings such as Gleason score, pathological T-stage (pT stage), and tumor volume were evaluated. We further used logistic regression and Cox proportional hazard regression analyses for assessing the association between tumor volume and PHI and for predicting biochemical recurrence. With an area under the curve (AUC) of 0.79, PHI is the most accurate predictor of a tumor volumes >0.5 cm^3^. Moreover, PHI correlates significantly with the tumor volume (*r* = 0.588), which is significantly different (*p* = 0.008) from the correlation of the Gleason score with tumor volume (*r* = 0.385). PHI correlates more strongly with the tumor volume than does the Gleason score. Using PHI improves the prediction of larger tumor volume and subsequently clinically significant cancer.

## 1. Introduction

Prostate cancer (PCa) is still one of the most frequent illnesses in men. Since the introduction of screening with prostate-specific antigen (PSA) in the early 1990s, the incidence of PCa has risen sharply, though the age at first diagnosis has been shifting toward younger patients with less advanced stages of PCa [1]. The number of deaths from PCa has been reduced in recent years, though not proportionally with the rise in incidence.

The degree of tumor expansion is the most important factor for both PCa prognosis and therapy [2]. The PCa tumor volume has a substantial influence on the course of the illness. Growth exceeding the capsule and lymph node metastases occurs mainly with larger tumors [3,4,5]. There is an approximately proportional relationship between the tumor volume and the stage of differentiation. Tumors that are smaller than 0.5 cm^3^ are classified as “insignificant”, since the tumor grows so slowly that there is a high probability that it will never reach a significant size before the death of the patient [6]. The probability of the existence of a tumor larger than 0.5 cm^3^ can be calculated, for example, with a nomogram [7].

The Prostate Health Index (PHI) is currently one of the most promising new markers of PCa. Initial studies have shown that PHI preferentially detects aggressive carcinomas and, due to its high specificity, can reduce the number of unnecessarily performed biopsies [8,9]. Furthermore, tumors larger than 0.5 cm^3^ have significantly higher PHI values than tumors with smaller volumes [10,11,12]. However, it is still not known whether PHI correlates continuously with tumor volume. A marker that is able to reliably predict the tumor volume would be advantageous for therapeutic decision-making. PHI and [−2]proPSA are currently the best available serum parameters for PCa detection [9]. At 90% sensitivity, the specificity of PHI for PCa detection is on average 31.6% [9]. The use of PHI in the framework of PCa screening reduces the use of biopsies by 15%–41%, in comparison to classic screening with only total prostate-specific antigen (tPSA) measurement [8,13]. Moreover, there is an association between PHI and carcinomas with unfavorable prognostic characteristics [12,14].

The use of PHI appears quite promising in patients with diagnosed PCa who are following an active surveillance strategy. Approximately 30%–37% of these tumors advance to stages requiring intervention [15,16]. With a PHI score >43, there is a 3.6 times higher risk for disease progression, according to a study by Hirama et al. [15]. Carcinomas with an elevated tendency for deterioration can be identified earlier this way and subjected to a definitive therapy. Overall, the integration of PHI into active surveillance appears to be also economically useful. The increased expenditures from this additional blood test would be outweighed since the reduction of unnecessary biopsies, doctor visits, and laboratory tests would reduce the costs in comparison to examinations based only on tPSA [17,18].

The aim of the present study was to determine if there is a continuous correlation between PHI and tumor volume over the entire range of values commonly seen.

## 2. Results

### 2.1. Clinicopathological Characteristics of the Study Cohort

All clinicopathological characteristics are summarized in Table 1. One-third of the patients (*n* = 65) had a well-differentiated tumor (Gleason < 7). A total of 131 patients had a poorly differentiated tumor (Gleason ≥ 7). Within this group, a Gleason 7 tumor was found in 57.6% of the patients and 9.7% of the patients had a Gleason 8 or 9, Table 1). About 80% of the patients had a locally confined tumor (pT ≤ 2c). An “insignificant” carcinoma (≤0.5 cm^3^, range 0.03 to 0.48 cm^3^) was found in 39 specimens (20%) and a “significant” tumor (>0.5 cm^3^, range 0.57 to 22.8 cm^3^) in 157 cases (80%).

### 2.2. PSA Parameters in Relation to Clinicopatholological Factors and Tumor Volume

In Table 2, the data of all four PSA derivatives and the prostate and tumor volumes are compiled in relation to the pathological Gleason score (well and poorly differentiated tumors) and the pathological stage (locally limited and advanced tumors). While percent free PSA (%fPSA) did not differ between the two tumor staging groups nor prostate volume both between the Gleason and staging groups, all other parameters were significantly different. The highest significance levela of *p* < 0.0001 were reached for the tumor volume and the percentage of tumor as well as for [−2]proPSA and PHI. In addition, Table 3 shows that all four serum parameters had significantly higher (tPSA, [−2]proPSA, PHI) or lower (%fPSA) values in patients with tumor volumes larger than 0.5 cm^3^, the so-called significant tumors. Additional details are summarized in the Supplementary Table S1.

**Figure 1:**
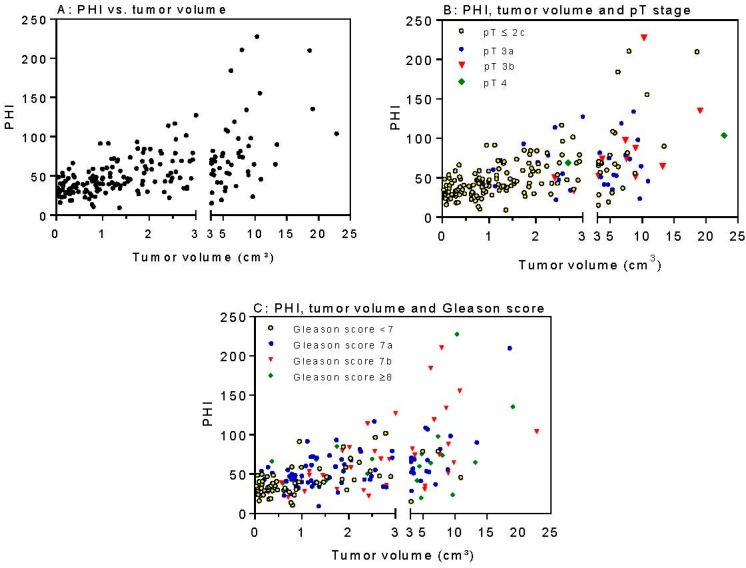
PHI values in relation to tumor volume, pT stage and Gleason score as indicated in (**A**–**C**).

The further associations between the clinicopathological variables and the PSA analytes were examined using correlation calculations and receiver-operating characteristi (ROC) curve analyses. Table 4 shows the correlations between the serum PSA parameters and the clinicopathological variables. PHI showed a significantly higher correlation to tumor volume than Gleason score (0.588 vs. 0.385; *p* = 0.008). Moreover, [−2]proPSA showed a significantly higher correlation with tumor volume than Gleason score (0.659 vs. 0.385, *p* = 0.0002). Interestingly, [−2]proPSA correlated significantly more with tumor volume than pT stage (0.659 vs. 0.522; *p* = 0.037). The correlation comparison between PHI and [−2]proPSA showed no difference (*p* = 0.25). PHI correlated significantly better with tumor volume than PSA (0.588 vs. 0.363; *p* = 0.004). The scatter plots of the PHI values in relation to the tumor volume as well as to the Gleason score categories and pT classifications are displayed in Figure 1A–C.

The areas under the ROC curves (AUCs) of the individual serum parameters are presented in Table 5. At 0.79, PHI reaches the greatest AUC value in differentiating tumor volumes ≤0.5 cm^3^ from those >0.5 cm^3^. In general, PHI is the best parameter to predict aggressive PCa with Gleason score (GS) ≥ 7 and locally advanced stages with ≥pT3a. Furthermore, PHI reached a significantly higher AUC than that of the pT stage in predicting a tumor volume of >0.5 cm^3^ (0.79 vs. 0.69; *p* = 0.04). Thus, to show the mutual influence and interdependence between all these variables, we performed a logistic regression analysis as a multivariable approach. For that purpose, the categorized Gleason score and pT stage in combination with PHI were analyzed to differentiate between the two tumor volume categories. All three variables showed a significant odds ratio (Gleason score: 6.61 with a 95% confidence interval of 2.72 to 16.1), *p*-value < 0.001; pT stage: (1.60 (1.01–2.55), *p* = 0.047; PHI: 1.04 (1.02–1.07, *p* = 0.002)). This combination distinctly increased the AUC value to 0.87 (0.82–0.91) indicating the improved prediction between the insignificant and significant tumor volume.

### 2.3. PHI and Tumor Volume as Independent Predictors of Biochemical Relapse

To evaluate the relationships between the conventional clinicopathological variables (Gleason score, pathological tumor stage, resection status) and age, tumor value, and PHI as predictors of the biochemical recurrence, we performed Cox regression analyses (Table 6). Both tumor volume and PHI were statistically significant predictors of a biochemical recurrence (follow-up time: 37 months (95% confidence interval 31 to 44)) in the univariate Cox regression model but also remained, despite the limited number of patients and events (*n* = 25) and the independent factors in the multivariable model (*p* = 0.048 and 0.0009).

## 3. Discussion

The use of PHI as a marker with high specificity for aggressive PCa and tumors with a low grade of differentiation has been proven [10,11,19]. In the present study, PHI correlates significantly with the tumor volume (*r* = 0.588). This is significantly stronger (*p* = 0.008) than the correlation of the Gleason score with tumor volume (*r* = 0.385). The hypothesis was supported by the significantly higher PHI value for tumor volumes >0.5 cm^3^ [10,11,12,14]. Furthermore, it seems plausible that the PHI values rise with increasing carcinoma volumes, since there is a proven relationship between tumor size and tumor differentiation [4,5,12,20]. Previous studies have shown that PHI can predict a tumor volume >0.5 cm^3^ even more precisely (AUC: 0.72–0.94) than Gleason score ≥7 (AUC: 0.64–0.74) or a pT-stage ≥ 3a (AUC: 0.72–0.85) [10,11,14,21,22]. The strong correlation between PHI and tumor volume found in this study describes a further partial aspect of the association of this marker with aggressive carcinomas.

PHI as a preoperative parameter shows significant explanatory power to predict a significant tumor volume in the prostatectomy preparation. In the present study, PHI reached a test strength (AUC) of 0.79 in the detection of clinical significant tumors. The portion of substantial tumors in our analysis was 80%. Comparable results for the test strength of PHI were reported in the work by Guazzoni et al. [11]. In 350 preparations, PHI detected tumors >0.5 cm^3^ with an AUC of 0.8. In that study, the portion of substantial tumors was 93%. Somewhat lower AUC values were found in the work from Tallon et al. [21]. In the framework of that study, 154 prostate preparations were examined. A volume >0.5 cm^3^ was found in 88% of the study participants. The calculated AUC value of PHI for the detection of substantial tumors was 0.72. In that study and in the present study, T3 tumors were included. In a study by Ferro et al., the AUC of PHI for the detection of substantial tumors was 0.94 [10]. The more advantageous results of that study might be explainable by the different composition of their study sample. On the one hand, that study limited itself to locally limited tumors (T1–T2); on the other hand, a comparatively smaller portion of the carcinomas (17%) had a substantial size (≥0.5 cm^3^). In the most recent study on 135 Asian patients by Chiu et al. [12], PHI reached a comparable high AUC of 0.82 to predict a tumor volume of >0.5 cm^3^, but only about half (52.3%) of all patients had a tumor volume >0.5 cm^3^. Since the portion of significant tumor volumes was 80% in our present study, comparability seems to be given more with the studies by Guazzoni et al. and Tallon et al. [11,21]. Furthermore, there is an association between the serum marker PHI and the pathological Gleason score. In our present study, PHI was the best parameter to detect tumors with a Gleason score ≥7 (AUC: 0.72) while the other parameters tPSA, %fPSA, and [−2]proPSA showed comparatively smaller AUCs of 0.64, 0.64, and 0.66, respectively. In other studies, the calculated AUC of PHI for the detection of tumors with a Gleason score ≥7 was between 0.67 and 0.74 [11,19,22]. In the work by Ferro et al. [10], an exceptionally high-test strength (AUC: 0.83) of PHI was found for the detection of tumors with a Gleason score ≥7. In addition, in this case, the clearly advantageous results from the research group of Ferro et al. [10] might be due to a different composition of the study group. Chiu et al. [12] provided only data for prediction of pathological Gleason score ≥7 or pathological pT3 stage and reached an AUC of exactly 0.8.

The relationship between PHI and biopsy Gleason score has already been investigated in numerous studies. According to a meta-analysis from 2014, PHI detects carcinomas with a Gleason ≥7 with an AUC of 0.90 [19]. That value is clearly above the calculated test strength of the present study for the detection of low differentiated tumors (AUC: 0.72). One possible source of such a large difference in results is that the biopsy Gleason score and the pathological Gleason score differ in many cases. The tissue samples of the core needle biopsy harbor a comparatively higher risk to incorrectly evaluate the Gleason grade more favorably than it actually is, since this technique of examination is inherently not capable of imaging the entire PCa. Since the Gleason score can differ according to the methods chosen, that meta-analysis is only conditionally comparable with the present study. Moreover, the processing of a prostatectomy preparation is the more precise method to characterize a PCa and, thus, to estimate the relation of Gleason score and PHI.

There is an association between PHI and the extensiveness of the local tumor. In the present study, PHI detected tumors with growth exceeding the capsule with an AUC of 0.70. The AUCs of other parameters were lower (tPSA: 0.64, %fPSA: 0.57, [−2]proPSA: 0.68). In other studies, the AUC for the detection of T3 tumors was reported as 0.69–0.72 [11,20], which is comparable to our study. In the study by Ferro et al. [10], PHI detected tumors exceeding the capsule with an AUC of 0.85.

In the present study, it is apparent that PHI shows the highest AUCs for the prediction of the tumor characteristics (0.79 for tumor volume, 0.72 for Gleason score ≥7, 0.70 for pT ≤ 3a). In other studies, it has been shown that PHI predicts the tumor volume more precisely than the Gleason score of the T-stage does [10,11,12,14,21]. Consequently, it can be assumed that the levels of PSA, fPSA, and [−2]proPSA (the components of the PHI) are influenced not only by the cell differentiation but likewise by the amount of carcinoma cells (tumor volume). According to the results of this study, it is conceivable that the tumor volume is the strongest determining factor. This is also supported by the high Pearson’s correlation factors of 0.588 and 0.478 for PHI with the tumor volume and the percentage of tumor, respectively. The correlation of PHI with the Gleason score (*r* = 0.309) and pT stage (*r* = 0.317) is weaker in our cohort. All these correlation data were unfortunately not provided by all previous studies. Furthermore, tumor volume (borderline significance with *p* = 0.048) and PHI (*p* = 0.0009) remained as independent factors in multivariable analysis to predict a biochemical recurrence (Table 6) beside the known pathological data pT stage and Gleason score. For example, the resection margin status could not reach significance (*p* = 0.27) in this Cox proportional hazard regression model, which emphasizes the importance of tumor volume and PHI.

The tumor volume is an important predictor of the further disease course and, correspondingly, should be kept in mind in planning therapy including focal therapy. Our method very precisely estimates tumor volume and has not been described before. Other earlier studies do not focus on methodological aspects. In the present work, the median tumor volume was 1.58 cm^3^, and the median PHI was 47.6. The results of this work show that a high PHI value is most likely connected with a large tumor volume, which could be a contraindication for a therapy approach of active surveillance. The integration of PHI into active surveillance could reduce the frequency of biopsies. Larger intervals between follow-up biopsies would be a clear advantage for the patient. Nevertheless, large-scale studies about this are needed in order to rule out the possibility that a lower frequency of biopsies in combination with regular PHI determinations leads to a worse outcome for the patient. The use of PHI in the preparation of a prostatectomy is likewise conceivable. High PHI values indicate a large tumor volume, which in turn makes the possibility of performing a nerve-sparing operation more improbable. Correspondingly, it could be advantageous to advise the patient of this aspect before operating. Future studies might be able to answer the question of whether there is a PHI limit value, above which a nerve-sparing operation becomes very improbable. This could lead to a more transparent risk prognosis before surgery.

The serum markers PHI and [−2]proPSA are accurate predictors of the tumor volume in prostatectomy preparations. Further, it was shown in the AUC analysis that PHI shows a stronger association to tumor volume than the established predictive parameters of the Gleason score and T-stage. It is important to integrate PHI into active surveillance of PCa patients, since an estimate of the tumor volume, as an important predictor of the illness progression, could support an adequate therapy decision.

## 4. Materials and Methods

### 4.1. Study Population

The study was approved by the Charité Ethics Committee. All patients provided written informed consent for this research study. Preoperative serum samples (tPSA, free PSA (fPSA), [−2]proPSA) were collected from 460 men who underwent radical prostatectomy between 2001 and 2014. The surgical approach was retroperitoneal, laparoscopic, or da Vinci based. The exclusion criteria were incomplete (*n* = 233) or missing pathological sections (*n* = 24), lack of appropriate serum samples (*n* = 2), or use of neoadjuvant therapies (*n* = 5) so that finally 196 patients were included in the analysis.

The study groups (Table 1) were defined as follows: carcinomas with a pathological GS of 4–6 were assembled into the GS < 7 group (well differentiated, *n* = 65) and carcinomas with a GS of 7–10 (*n* = 131) were assembled into the GS ≥ 7 group (poorly differentiated). The stage “locally limited” (*n* = 154) included tumors with pathological T-stage (pT stage) 2a, 2b, and 2c. The stage “locally advanced” included tumors in the pT-stages 3a, 3b, and 4 (*n* = 42). The volume of the tumors was divided into two categories: “insignificant” (≤0.5 cm^3^, *n* = 39) and “significant” (>0.5 cm^3^, *n* = 157) according to the Epstein criteria [23].

The prognostic potential of the tumor volume in relation to the other clinicopathological parameters including PHI was assessed to predict tumor recurrence after radical prostatectomy. The conventional criterion of biochemical recurrence of increased PSA after surgery was used. Biochemical recurrence was defined as the first postoperative PSA value of >0.1 ng/mL following a nadir PSA level after surgery and confirmed by persistent increased PSA values >0.1 ng/mL. The interval between radical prostatectomy and biochemical recurrence was calculated in months as time to the development of this event, while patients without biochemical recurrence were censored at the last follow-up visit.

### 4.2. Pathology Assessment

After the macroscopic assessment, the preparations were weighed and measured in three planes (apico-basal, horizontal, ventro-dorsal). The prostates were sliced first medially along the urethra and then the apex and base were cut off. Both mid-pieces, the apex, and the base were each divided into approximately 5 mm thick tissue blocks and then embedded in paraffin. The entire paraffin blocks were sliced by machine into 2–4 µm thin sections and then mounted onto specimen slides. They were then stained with hematoxyline and eosin (H&E). The prostatectomy preparations of this study had a mean (range) of 28 (10–63) sections; in total, over 5500 H&E sections were examined microscopically.

### 4.3. Serological Diagnosis and Volumetric Analysis

The preoperative determination of serum tPSA, fPSA, and [−2]proPSA was performed on the fully automated immunoassay device Access^®^ (Beckman Coulter, Brea, CA, USA) as described before [24]. PHI was calculated according to the equation [−2]proPSA/fPSA × √PSA.

All tissue sections were examined under a microscope, and carcinomas were outlined in color. The appraisal of the preparation was performed together with an experienced pathologist (EK).

**Figure 2:**
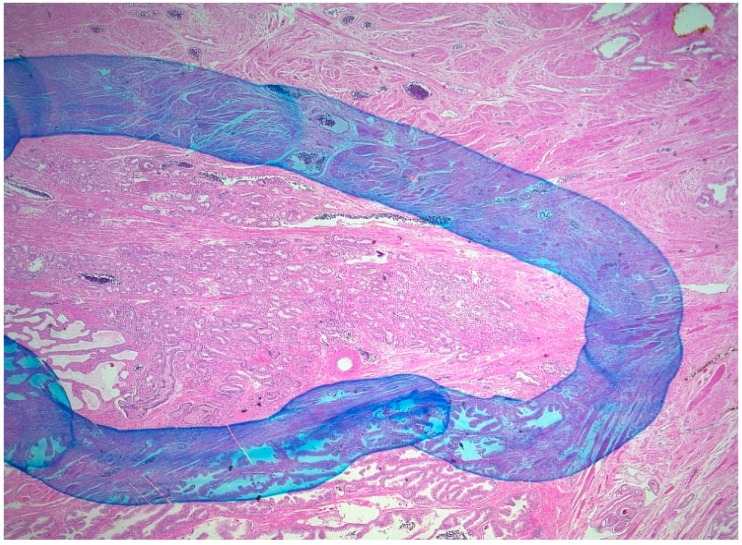
Outlining of the prostate carcinoma. (Hematoxyline-eosin stain; magnification: 20×).

As Figure 2 illustrates, carcinomas were surrounded with a hand-drawn line. The surfaces of the prostate and the surface of the tumor on the H&E sections were identified by means of a gridwork screen (3 mm × 3 mm). The gridwork screen was placed on the slides, and the surfaces were enumerated. The boxes were evaluated as “1”, “0.5”, and “0” when they were covered with clearly more than 50%, approximately with 50%, or clearly less than 50% by tumor, respectively. For each H&E section, the number of boxes for prostate and for cancer were recorded separately. Periprostatic soft tissue and intraprostatic parts of the seminal vesicle were excluded from the evaluation. The volume of the prostate was calculated with the aid of the ellipsis formula (π/6 × height × width × depth). The percentage portion of the carcinoma was calculated as follows: %tumor = (total number of tumor boxes/total number of prostate boxes) × 100. The tumor volume was then calculated with the following formula: tumor volume = (%tumor × prostate volume)/100.

### 4.4. Statistical Analysis

All statistical analysis was performed with SPSS 22.0 (IBM Corporation; Armonk, NY, USA) and MedCalc 16.8.4 (MedCalc software bvba, Ostend, Belgium). Several tests were performed (Mann–Whitney *U*-test, Kruskal–Wallis test, Pearson correlation coefficient, logistic regression, and Cox hazard regression). ROC analysis was used for estimating the AUCs. A *p*-value < 0.05 was considered statistically significant.

## Figures and Tables

**Table 1:** Clinicopathological data (median and range) of the study cohort.

Variable	Median (Range)	Mean ± S.D.
Age (years)	71 (49–85)	70 ± 7
Prostate volume (cm^3^)	36.7 (9.2–264)	42.9 ± 27.1
Tumor volume (cm^3^)	1.57 (0.03–22.8)	2.76 ± 3.47
Percentage of tumor volume	4.3 (0.02–80.4)	7.8 ± 10.6
	**Number (Percent)**	
Pathological tumor stage		
≤2c	154 (78.6)	
3a	29 (14.8)	
3b	11 (5.6)	
4	2 (1.0)	
Gleason Score		
<7	65 (33.2)	
7a (3 + 4)	77 (39.2)	
7b (4 + 3)	36 (18.4)	
≥8	18 (9.2)	
Resection margin status		
R0	143 (73.0)	
R1	50 (25.5)	
Rx	3 (1.5)	

**Table 2:** Prostate volume, tumor volume, and prostate-specific antigen (PSA) analytes (median and range) in dependence on Gleason score and pathological tumor stage.

Variable	All Patients	Gleason Score <7 ≥7		*p*-Value	pT Stage ≤2c ≥3		*p*-Value
	(*n* = 196)	(*n* = 65)	(*n* = 131)		(*n* = 154)	(*n* = 42)	
Prostate volume (cm^3^)	36.7 (9.2–264)	36.8 (17.6–167)	36.1 (9.2–264)	0.568	35.3 (14.7–167)	39.8 (9.2–264)	0.295
Tumor volume (cm^3^)	1.58 (0.03–22.8)	0.66 (0.03–10.9)	2.26 (0.04–22.8)	<0.0001	1.21 (0.03–18.6)	4.36 (0.87–22.8)	<0.0001
Percentage of tumor (%)	4.3 (0.02–80.4)	1.4 (0.02–40.3)	6.6 (0.1–80.4)	<0.0001	3.1 (0.02–50.0)	10.0 (3.1–80.4)	<0.0001
tPSA (ng/mL)	5.0 (0.7–61.6)	4.2 (0.7–17.7)	5.4 (0.7–61.6)	<0.001	4.6 (0.7–61.6)	5.6 (1.8–32.6)	0.005
%fPSA (%)	12.3 (4.0–76.6)	14.4 (4.9–36.9)	11.3 (4.0–76.6)	<0.001	12.7 (4.0–76.6)	11.5 (4.9–28.2)	0.154
[−2]proPSA (pg/mL)	12.3 (2.3–117)	10.0 (2.3–46.7)	14.2 (3.2–117)	<0.0001	11.7 (2.3–117)	17.4 (3.7–58.2)	0.0005
Prostate Health Index (PHI)	47.6 (9.3–228)	37.5 (10.5–102)	55.3 (9.3–228)	<0.0001	43.4 (9.3–211)	67.1 (22.3–228)	<0.0001

Statistical significances were calculated using the Mann–Whitney *U*-test.

**Table 3:** PSA parameters (medians and ranges) in dependence on the tumor volume.

Variable	Tumor Volume		*p*-Value
	≤0.5 cm^3^ (*n* = 39)	>0.5 cm^3^ (*n* = 157)	
tPSA (ng/mL)	2.8 (0.7–10.8)	5.4 (0.7–61.6)	<0.0001
%fPSA	15.8 (6.0–35.0)	11.7 (4.0–76.6)	0.0006
[−2]proPSA (pg/mL)	9.4 (2.3–32.0)	13.6 (3.2–117)	<0.0001
PHI	32.9 (16.1–66.4)	53.7 (9.3–228)	<0.0001

Statistical significance calculated using the Mann–Whitney *U*-test.

**Table 4:** Pearson correlation of the serum parameters and the pathology findings.

Parameter	Tumor Volume (cm^3^)	Tumor (%)	Gleason Score (≤6, 7a, 7b, ≥8)	pT Stage (≤2c, 3a, 3b, 4)
tPSA	0.363 (<0.0001)	0.287 (<0.0001)	0.153 (0.032)	0.237 (0.0008)
%fPSA	−0.101 (0.158)	−0.145 (0.043)	−0.157 (0.028)	−0.071 (0.324)
[−2]proPSA	0.659 (<0.0001)	0.389 (<0.0001)	0.246 (0.0005)	0.344 (<0.0001)
PHI	0.588 (<0.0001)	0.478 (<0.0001)	0.309 (<0.0001)	0.317 (<0.0001)
Gleason Score	0.385 (<0.0001)	0.373 (<0.0001)	-	0.397 (<0.0001)
pT Stage	0.522 (<0.0001)	0.486 (<0.0001)	0.397 (<0.0001)	-

The table presents the *r* values, with the *p*-values in parentheses.

**Table 5:** Calculated areas under the receiver operating characteristic (ROC) curves of the serum parameters (left column) to predict tumors with particular characteristics (top row). The 95% confidence intervals of the area under the curves (AUCs) are given in parentheses.

Parameter	Gleason Score ≥ 7 vs. <7	pT Stage ≥3a vs. ≤2c	Tumor Volume >0.5 vs. ≤0.5 cm^3^
tPSA	0.64 (0.56–0.73)	0.64 (0.55–0.73)	0.74 (0.65–0.83)
%fPSA	0.64 (0.56–0.72)	0.57 (0.48–0.67)	0.68 (0.58–0.77)
[−2]proPSA	0.66 (0.59–0.74)	0.68 (0.58–0.77)	0.72 (0.64–0.81)
PHI	0.72 (0.65–0.80)	0.70 (0.62–80.0)	0.79 (0.72–0.86)

**Table 6:** Cox proportional hazard regression analyses of tumor volume and PHI in relation to conventional clinicopathological variables for predicting biochemical recurrence after radical prostatectomy.

Variable ^a^	Univariable Analysis		Multivariable Analysis ^b^	
	Hazard Ratio (95% CI ^c^)	*p-*Value	Hazard Ratio (95% CI)	*p*-Value
Age (continuous)	1.05 (0.98–1.12)	0.159	not included	
pT stage	4.27 (1.91–9.56)	0.0004	3.92 (1.60–9.56)	0.003
Gleason Score	1.97 (1.22–3.18)	0.006	1.91 (1.01–3.57)	0.042
Margin resection status	3.34 (1.52–7.35)	0.003	1.65 (0.67–4.01)	0.273
Tumor volume	1.12 (1.01–1.24)	0.047	0.83 (0.68–0.99)	0.048
PHI	1.02 (1.01–1.03)	<0.0001	1.02 (1.01–1.03)	0.0009

^a^ Age and tumor volume were used with their continuous values, the pathological factors as categorized data as indicated in the previous tables; ^b^ The multivariable analysis included all variables with *p-*values < 0.10 obtained in the univariable analysis; ^c^ CI = confidence interval.

## Supplementary Materials

Supplementary materials can be found at www.mdpi.com/1422-0067/18/3/488/s1.
