# Supplementary material for: Does the Prostate Health Index Depend on Tumor Volume?—A Study on 196 Patients after Radical Prostatectomy
Source: Int J Mol Sci. 2017 Feb 24;18(3):488. doi: 10.3390/ijms18030488 (PMC5372504; doi:10.3390/ijms18030488)
Supplement: Supplementary file 1 [file ijms-18-00488-s001.pdf]

Supplementary Materials: Does the Prostate Health Index Depend on Tumor Volume?—A Study on 196 Patients after Radical Prostatectomy

Frank Friedersdorff, Britt Groß, Andreas Maxeiner, Klaus Jung, Kurt Miller, Carsten Stephan, Jonas Busch and Ergin Kilic

Table S1. Relevant PSA derivatives (median and ranges) in relation to tumor volume, Gleason Score and pathological tumor stage.

Variable	Tumor Volume		Gleason Score				pT Stage			
	**≤0.5 cm³**	**>0.5 cm³**	**≤7**	**7a**	**7b**	**≥8**	**≤2c**	**3a**	**3b**	**4**
	**(*n* = 39)**	**(*n* = 157)**	**(*n* = 65)**	**(*n* = 77)**	**(*n* = 36)**	**(*n* = 18)**	**154**	**29**	**11**	**2**
tPSA (ng/mL)	2.8 (0.7–10.8)	5.4 (0.7–61.6)	4.2 (0.7–17.7)	4.7 (1.4–27.8)	5.9 (0.7–61.6)	6.1 (1.8–18.3)	4.6 (0.7–61.6)	5.0 (1.8–17.7)	7.2 (3.3–14.9)	19.7 (6.7–32.6)
%fPSA	15.8 (6.0–35.0)	11.7 (4.0–76.6)	14.4 (4.9–36.9)	11.9 (4.1–76.6)	10.4 (4.0–30.0	11.3 (6.2–19.6)	12.7 (4.0–77.6)	11.7 (4.9–28.2)	10.8 (6.2–19.4)	16.0 (11.3–20.7)
[-2]proPSA (pg/mL)	9.4 (2.3–32.0)	13.6 (3.2–117)	10.0 (2.3–46.7)	13.6 (3.2–117)	14.8 (3.4–57.3)	17.5 (4.6-58.2)	11.7 (2.3–117)	15.1 (3.7–51.5)	27.0 (8.3–58.2)	50.3 (44.9–55.7)
PHI	32.9 (16.1–66.4)	53.7 (9.3–228)	37.5 (10.5–101)	52.5 (9.3–210)	66.8 (20.7–211)	65.5 (19.4-228)	43.4 (9.3–211)	60.4 (22.3–134)	74.4 33.6–228)	86.6 (69.4–104)
